# Multi-Chain Semi-Markov Analysis of Intrapartum Cardiotocography

**DOI:** 10.1109/EMBC48229.2022.9871665

**Published:** 2022-07

**Authors:** Johann Vargas-Calixto, Yvonne Wu, Michael Kuzniewicz, Marie-Coralie Cornet, Heather Forquer, Lawrence Gerstley, Emily Hamilton, Philip A. Warrick, Robert E. Kearney

**Affiliations:** Department of Biomedical Engineering, McGill University, Montreal, QC H3A 2B4, Canada.; University of California, San Francisco, CA 94158, USA.; Kaiser Permanente, Oakland, CA 94612, USA.; University of California, San Francisco, CA 94158, USA.; Kaiser Permanente, Oakland, CA 94612, USA.; Kaiser Permanente, Oakland, CA 94612, USA.; McGill University, Montreal, QC H3A 2B4, Canada, and with PeriGen Inc., Montreal, QC H4Z1E8, Canada.; McGill University, Montreal, QC H3A 2B4, Canada, and with PeriGen Inc., Montreal, QC H4Z1E8, Canada.; Department of Biomedical Engineering, McGill University, Montreal, QC H3A 2B4, Canada.

## Abstract

Visual assessment of the evolution of fetal heart rate (FHR) and uterine pressure (UP) patterns is the standard of care in the intrapartum period. Unfortunately, this assessment has high levels of intra- and inter-observer variability. This study processed and analyzed FHR and UP patterns using computerized pattern recognition tools. The goal was to evaluate differences in FHR and UP patterns between fetuses with normal outcomes and those who developed hypoxic-ischemic encephalopathy (HIE). For this purpose, we modeled the sequence of FHR patterns and uterine contractions using Multi-Chain Semi-Markov models (MCSMMs). These models estimate the probability of transitioning between FHR or UP patterns and the dwell time of each pattern. Our results showed that in comparison to the control group, the HIE group had: (1) more frequent uterine contractions during the last 12 hours before birth; (2) more frequent FHR decelerations during the last 12 hours before birth; (3) longer decelerations during the last eight hours before birth; and (4) shorter baseline durations during the last five hours before birth. These results demonstrate that the fetuses in the HIE group were subject to a more stressful environment than those in the normal group.

## I. Introduction

During labor, clinicians assess fetal wellbeing using cardiotocography (CTG): the monitoring of fetal heart rate (FHR) and uterine pressure (UP). Clinicians analyze the FHR and UP signals using a set of rules designed to predict whether a fetus is at risk of fetal acidemia or will have a normal outcome [1]. This assessment identifies and analyzes stereotypical FHR patterns, such as the FHR baseline, accelerations, and decelerations. Unfortunately, the visual assessment of these patterns has large intra- and inter-observer variability [2]. Furthermore, the guidelines for assessing FHR signals are not very sensitive for detecting worrisome patterns. As a result, up to 80% of FHR tracings are evaluated as “indeterminate risk” [3].

There is an increasing availability of computerized tools to process CTG signals more objectively and reproducibly than visual assessment. PeriCALM Patterns is a specialized software that processes and identifies the main FHR patterns and the presence of uterine contractions [4, 5]. Furthermore, recent studies have applied machine learning (ML) methods to assess fetal pathologies from CTG data automatically [6]. The early detection of fetal compromise in the intrapartum period can lead to preventive interventions such as emergency Cesarean delivery. When these interventions are carried out promptly, they can prevent the development of severe fetal acidemia and subsequent hypoxic-ischemic encephalopathy (HIE). Thus, there is an increasing interest in using ML to reduce the risk of HIE in labor.

Importantly, CTG signals are nonstationary during the intrapartum period. As labor progresses, uterine contractions become larger and more frequent. Uterine contractions cause short periods of fetal hypoxia, which cause FHR decelerations [7]. Hence, the properties of FHR and UP signals are time-varying throughout labor. For that reason, we must use time-varying analysis methods during the intrapartum period. Previously, we examined the temporal evolution of FHR variability properties [8]. The current study analyzes the time-varying sequential behavior of the FHR patterns and uterine contractions, which is the focus of bedside clinical assessment. Onu et al. proposed that the sequential behavior of physiological data could be analyzed using Multi-Chain Semi-Markov models (MCSMMs) [9]. These models generate estimates of the time-varying probability of switching between FHR patterns. The objective of our study is to quantify the differences in the MCSMMs parameters of CTG between fetuses with normal outcomes and those that developed HIE.

## II. Clinical Data

This study used data collected in 15 Kaiser Permanente Northern California hospitals between 2011 and 2019. Our database comprises clinical and CTG data of 246,968 singleton births with a gestational age of at least 35 weeks. We analyzed CTG signals in the last 12 hours before delivery, which were sampled at 4 Hz. In this study, HIE was diagnosed by a blood pH < 7 or base deficit > 10 mmol/L accompanied by clinical evidence of encephalopathy. The current study was limited to subjects who had a cord blood gas or neonatal blood gas available within the first 120 minutes of birth. Also, we limited our analysis to individuals born vaginally to avoid cases where Cesarean delivery interrupted the natural progression of labor. These selections generated a group of 21,717 individuals with a normal outcome and 161 individuals that developed HIE. Finally, this study made no attempt to account for differences in medications administered during labor.

## III. Methods

### A. Identification of CTG Patterns

CTG signals were processed using PeriCALM Patterns, a software developed by PeriGen Inc. to identify characteristic CTG events during the intrapartum period. It uses long short-term memory (LSTM) networks to identify FHR events and logistic regression to detect UP contractions [4, 5].

#### 1) Preprocessing

PeriCALM Patterns applies linear interpolation to fill gaps shorter than 60 samples (15 s). This prepares the signals for low-pass, high-pass, median, and FHR Karhunen-Loève filtering steps, which generate inputs for the respective UP and FHR event detectors.

#### 2) Identification of CTG Patterns

PeriCALM Patterns identifies the following:

- FHR Baseline (BAS): FHR segments typically in the range of 110 – 160 bpm, with a peak-to-peak variability of 5 – 15 bpm.
- FHR Acceleration (ACC): segments where the FHR increases more than 15 bpm for more than 15 seconds and then returns to baseline.
- FHR Deceleration (DEC): segments where the FHR decreases more than 15 bpm for more than 15 seconds and then returns to baseline.
- Uninterpretable FHR (NOI): noisy or missing segments in the FHR or artifacts that are not repaired by PeriCALM Patterns. We removed these segments from the analysis.
- Uterine Contraction (CON): segments where the UP amplitude increases before returning to resting tone.
- Resting Interval (RIN): UP when not in CON.

During labor, clinicians base their assessment of fetal wellness on the visual examination of CTG patterns. In contrast, PeriCALM Patterns provides an objective and reproducible approach to CTG pattern analysis.

### B. Analysis of the sequence of events

Fig. 1 shows the output of PeriCALM Patterns for a 20-minute window of intrapartum CTG. NOI events, which were discarded, are shown as gaps in the FHR signal. We did not consider transitions interrupted by NOI longer than 15s, the minimum length of an FHR event. For example, the transition from DEC to BAS at 90 minutes was interrupted by a gap and not counted. However, if a gap was shorter than 15 seconds, we assumed that no other event intervened and counted that transition. Similarly, gaps shorter than 15 seconds were ignored in estimating the dwell time of each event.

### C. Multi-Chain Semi-Markov Models (MCSMMs)

We based our analysis on the methods proposed by Onu et al. [9], who used MCSMMs to model the time-varying behavior of sequential physiological processes. MCSMMs model the evolution of Semi-Markov chains as a function of time. To do so, we divided the CTG signals into 36 nonoverlapping, 20-minute epochs. We discarded any epoch with less than 80% valid samples. The epoch starting N minutes before birth will be referred to as epoch-N. There were three possible FHR states (BAS, DEC, and ACC) and two possible UP states (RIN and CON) within each epoch. We estimated the transition probabilities for each epoch as:

(1) $$p(i\to j)=\frac{n(i\to j)}{\sum_{j=1}^{3}n(i\to j)},$$

where n(i→j) is the number of times that state i transitioned into state j. Thus, p(i→j) measures the proportion of state i transitions towards state j. These probabilities did not account for the self-transitions. Instead, we estimated the dwell-time, the probability distribution of the time spent in one state before transitioning. Thus, the transition probabilities are defined only during transitions and not on a sample-by-sample basis. The state transitions and dwell times are shown for the FHR in Fig. 2.A, and for UP in Fig. 2.B. Transition probabilities and dwell times were computed separately for each epoch.

In the FHR model, there were only two transitions possible for each state. Thus, the time-varying transition matrices were summarized by three of the six transition probabilities:

(2) $$p_{BAS\to DEC}=1-p_{BAS\to ACC},$$

(3) $$p_{DEC\to ACC}=1-p_{DEC\to BAS},\text{and}$$

(4) $$p_{ACC\to DEC}=1-p_{ACC\to BAS}$$

We estimated the 2.5th, 50th, and 97.5th percentile of these probabilities from 1,000 bootstrap samples determined by randomly sampling with replacement from the array of transitions and estimating the transition probabilities. In the UP model, each state had only one possible transition. Consequently, we focused on the RIN and CON dwell times.

To analyze trends of the dwell times, we removed values that were smaller than the 2.5th percentile and those that were larger than the 97.5th percentile. This was to prevent outliers from biasing the estimates of the trend. Then, we estimated the expected value of the remaining samples and their 2.5th, 50th, and 97.5th percentiles using 1,000 bootstrap samples.

### D. Statistical Analysis

We compared the differences between the two groups in terms of the transition probabilities and the dwell times. We estimated the 95% confidence interval (CI) of the transition probabilities as their 2.5th – 97.5th percentile range. Thus, differences were deemed statistically significant when the 95% CI of the transition probabilities did not overlap.

In contrast, dwell times are described by continuous probability distributions. Consequently, we used the two-sample Kolmogorov-Smirnov (KS) test to assess the statistical significance of the differences between the groups. We rejected the null hypothesis that the distributions were the same with a confidence of 95%.

## IV. Results

### A. Preprocessing of CTG Patterns

The processing of CTG signals is challenging due to their low signal-to-noise ratio and the presence of frequent artifacts. PeriCALM Patterns was an essential tool in the preprocessing and event detection of FHR and UP signals. Fig. 1 shows an example CTG tracing with identified patterns during epoch-100. PeriCALM Patterns automatically identified these patterns in the 21,877 available records. There were long gaps in some signal records. PeriCALM Patterns identified and flagged these NOI segments, permitting their removal from consideration. Fig. 3 illustrates the effect of noise and artifacts in CTG. It shows the percentage of valid epochs as a function of time for both groups. The number of epochs with at least 80% of valid samples increased with time and decreased drastically in epoch-20.

### B. Multi-Chain Semi-Markov Models

Fig. 4 summarizes the behavior of the transition matrices as a function of time before delivery for the normal and HIE groups. Fig. 4.A shows that the probability of a baseline to decelerations transition, $p_{BAS\to DEC}$, increased as delivery approached for both groups. Moreover, $p_{BAS\to DEC}$ was significantly higher throughout most of the 12 hours for the HIE group except for final epoch-40 and epoch-20. Fig. 4.B shows that there was little if any difference in the probability of transition from deceleration to acceleration, $p_{DEC\to ACC}$, between the two groups. Finally, Fig. 4.C shows that the transition probability from acceleration to deceleration, $p_{ACC\to DEC}$, was similar for the two groups and increased in the last 4 hours before delivery.

Fig. 5 shows the temporal evolution of the expected value of the dwell times of baseline, deceleration, and acceleration for the normal and HIE groups. Fig. 5.A shows that baseline dwell time decreased with time for both groups. It also shows that the baseline dwell time was initially larger in the HIE group than in the normal group. However, in the last 4 hours of labor it became shorter in the HIE group. Fig. 5.B shows that the deceleration dwell time slightly increased as delivery approached in both groups. Moreover, it was longer for the HIE group from 8 hours before delivery. Finally, Fig. 5.C shows that the acceleration dwell time did not vary with time and was similar between the two groups.

Lastly, Fig. 6 shows the dwell times of the contractions and resting intervals. Fig. 6.A shows that the contraction dwell time did not vary much with time, and it was slightly larger in the HIE group during the last 2 hours. Finally, Fig. 6.B shows that the resting interval dwell time diminished over time in both groups and that the HIE group had consistently shorter resting intervals than the controls throughout the 12 hours.

## V. Discussion

Our results showed significant differences in the CTG patterns of fetuses that had a normal outcome and those that developed HIE. The resting interval dwell time became shorter as delivery approached, with contraction dwell time relatively constant, implying that contractions became more frequent. Moreover, the resting interval dwell time was shorter in the HIE than in the normal group. Uterine contractions cause short periods of fetal hypoxia. If sustained for long periods without enough time for recovery, they can overwhelm the fetal protective mechanisms, leading to severe fetal acidemia and subsequent HIE [7]. We showed that the fetuses in the HIE group endured more frequent contractions than those with normal outcomes.

Our results also showed that fetuses that developed HIE had a higher baseline to deceleration transition probability $\left(p_{BAS\to DEC}\right)$, and more prolonged deceleration dwell time than those with normal outcomes. Conversely, the complementary probability $p_{BAS\to ACC}$ decreased with time. Also, the baseline dwell time became shorter in the HIE group as delivery approached. More importantly, these differences were significant several hours before labor. The dwell times showed differences 4 hours before labor for the baseline and 8 hours for deceleration.

It is crucial to highlight the early differences showed by MCSMMs between fetuses that developed HIE and those with normal outcomes. The resting interval dwell time and the $p_{BAS\to DEC}$ showed differences as early as 12 hours before birth, whereas most of the other parameters showed differences up to 4 hours before birth. HIE is associated with longer labors, slower progress and more frequent contractions and decelerations [10]. These conditions have compounding adverse effects on the fetus. Strong contractions restrict maternal blood flow through the myometrial vessels to the placenta and hence oxygen delivery to the fetus. Frequent contractions lessen the resting interval, the time for myometrial relaxation and placental reperfusion and for fetal compensatory mechanisms to achieve homeostasis before the next contraction begins. Decelerations reflect compensatory fetal mechanisms to reduce cardiac oxygen consumption in the presence of hypoxemia. However, deep, and frequent decelerations with long duration are associated with fetal hypotension, a sign of fetal decompensation [11]. Fetal hypotension and failure to maintain oxygen delivery to vital organs is associated with fetal brain injury. The evolution of the FHR and UP measures that we considered may help determine when fetal decompensation is imminent, and interventions such as Cesarian sections are appropriate.

## VI. Conclusion

We showed that computerized tools such as PeriCALM Patterns could be used to evaluate CTG patterns objectively Our results show that MCSMMs are a promising approach to the analysis of CTG and the early detection of fetuses at risk of developing HIE. Thus, we demonstrated that there were significant differences in some MCSMM parameters as early as 12 hours before birth, encouraging their subsequent use in machine learning. In future work, we will integrate these parameters with other FHR variability measures such as power spectral density and nonlinear features [8] in a machine learning procedure aimed at predicting the increased risk of developing HIE from CTG.

## Figures and Tables

**Figure 1.**
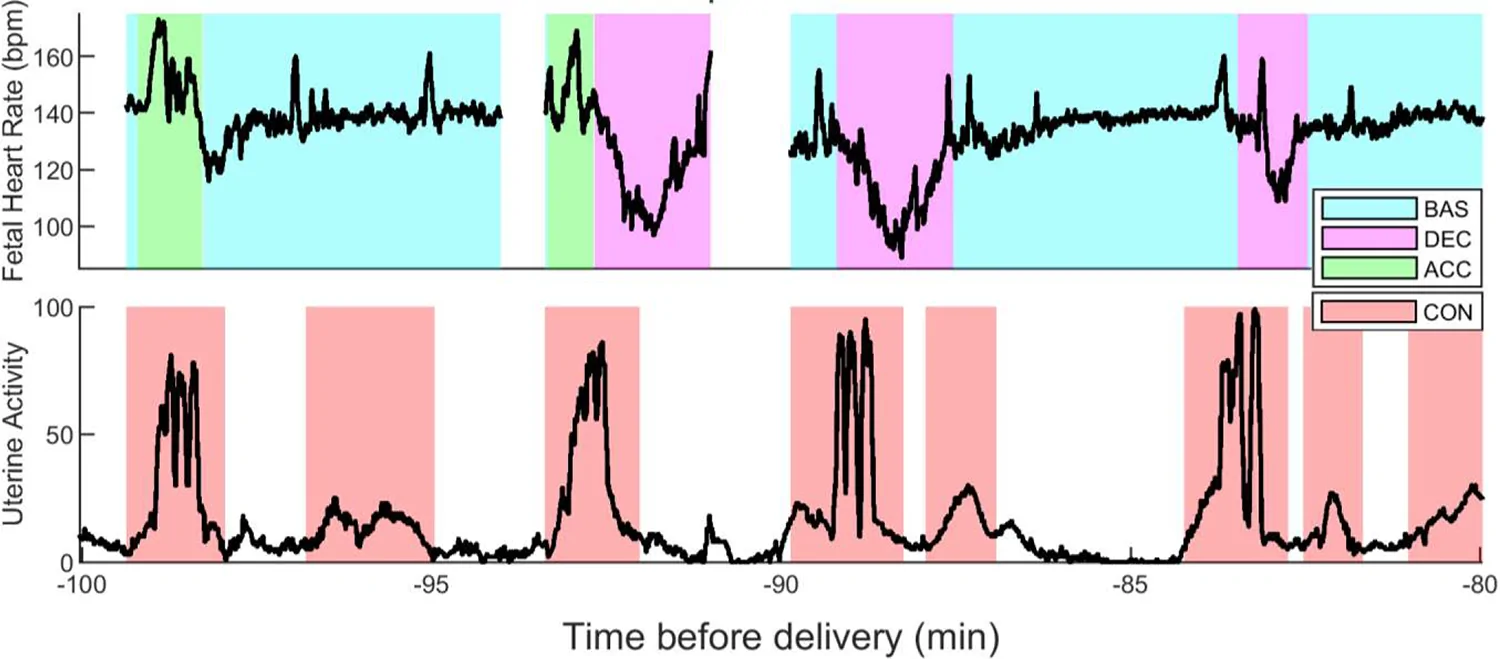
FHR and UA patterns identified using PeriCALM Patterns for one subject in a window of 20 minutes starting 100 minutes before birth.

**Figure 2.**
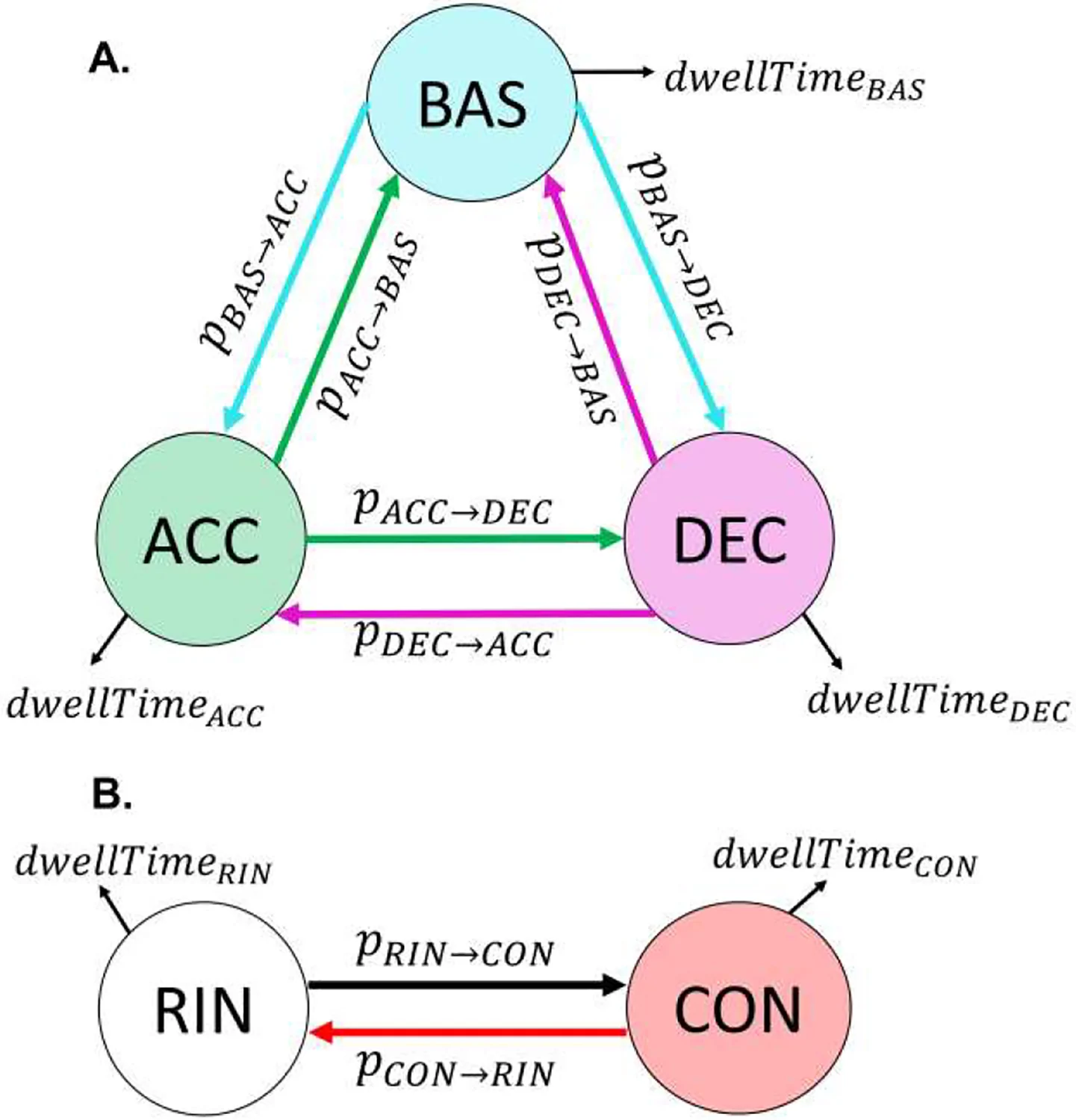
Diagram of the transitions probabilities and dwell times for the (A) fetal heart rate and (B) uterine pressure patterns

**Figure 3.**
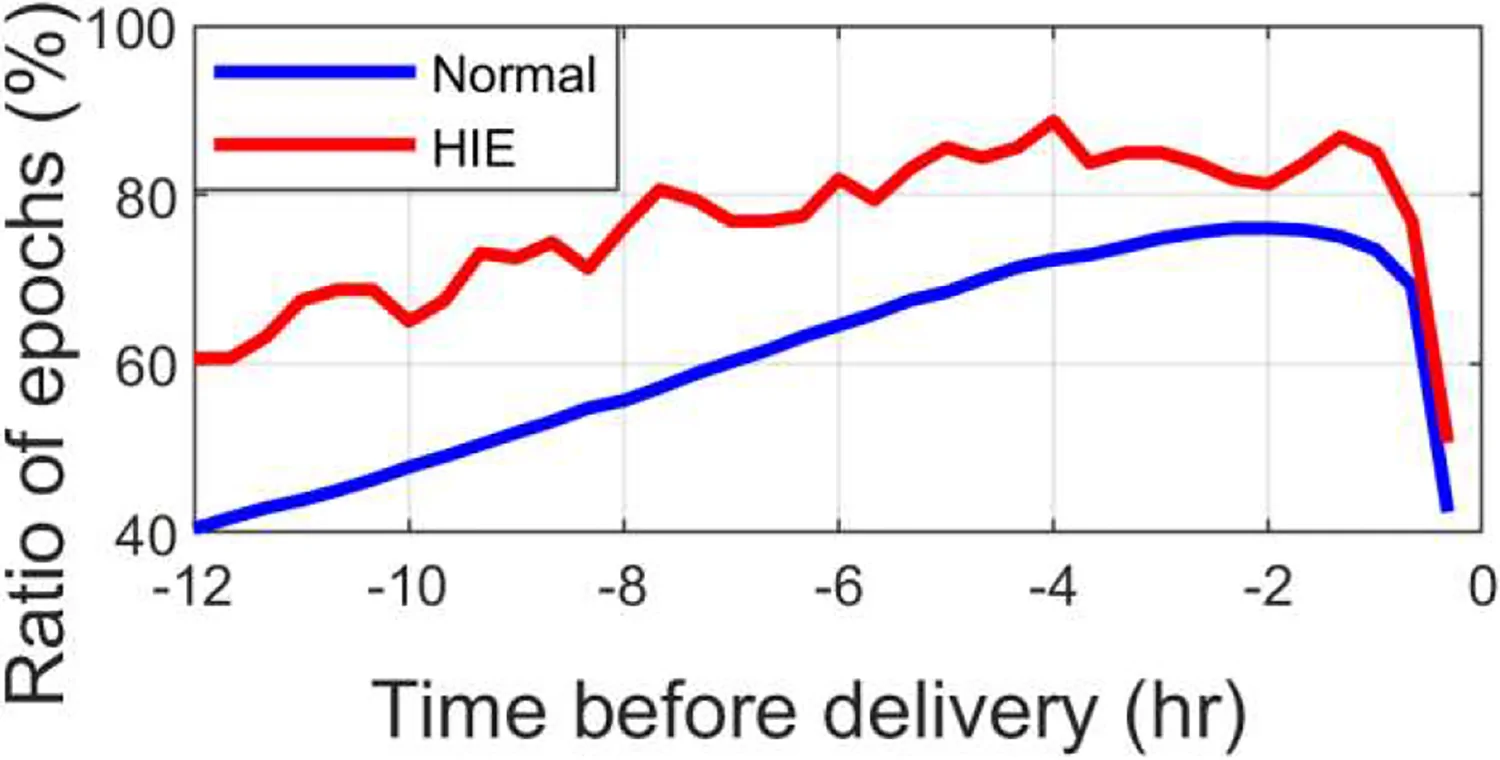
Percentage of valid epochs as a function of time before delivery for the normal (blue) and HIE (red) groups.

**Figure 4.**
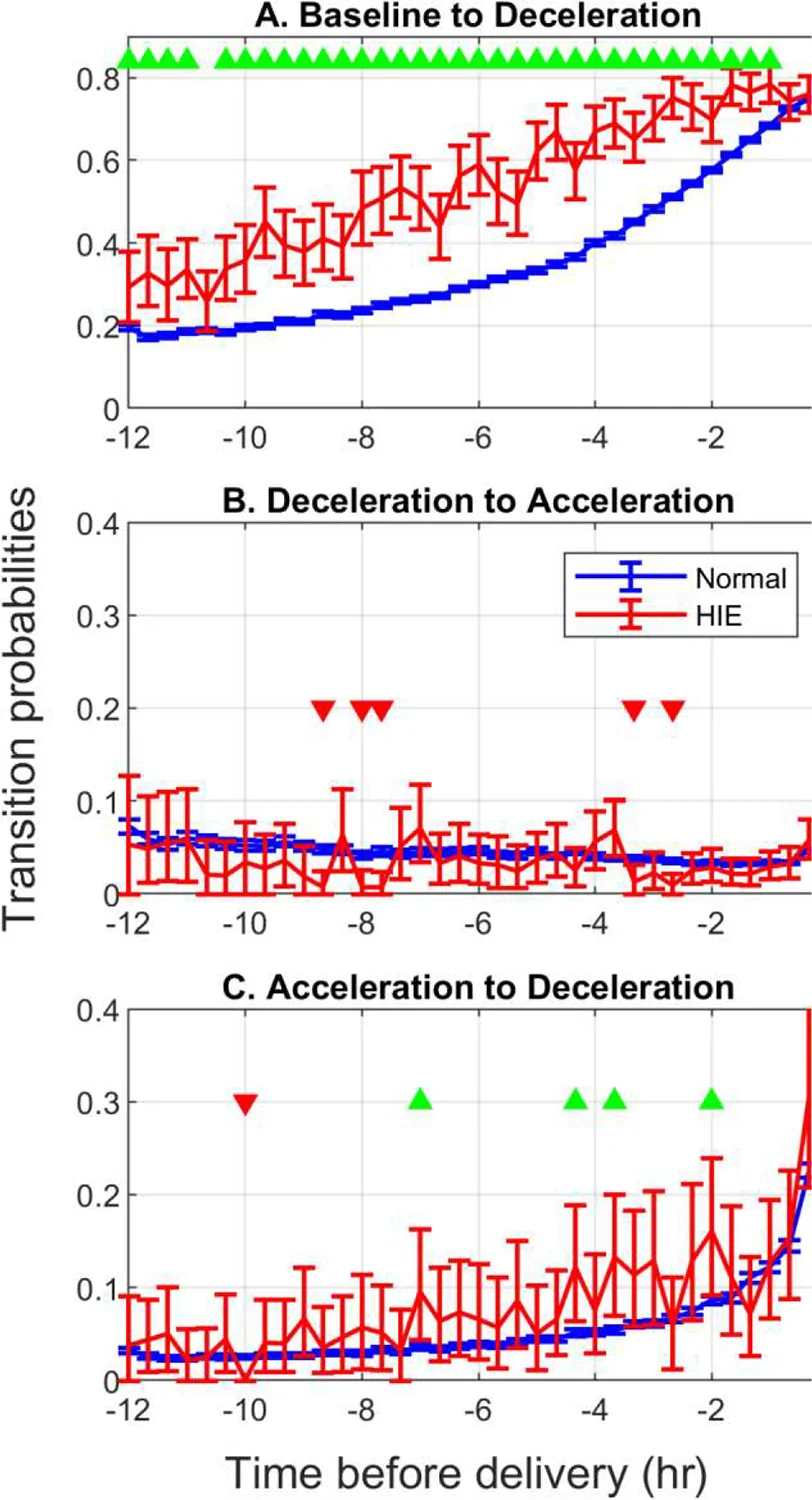
The 50^th^ percentile of (A) baseline to deceleration $p_{BAS\to DEC}$, (B) deceleration to acceleration $p_{DEC\to ACC}$, and (C) acceleration to deceleration $p_{ACC\to DEC}$ transition probabilities as functions of time before delivery for the normal (blue) and HIE (red) groups. The error bars show the 2.5^th^ – 97.5^th^ percentile range. Triangles indicate significant differences and whether the estimates were larger (green upwards) or smaller (red downwards) for HIE than for controls.

**Figure 5.**
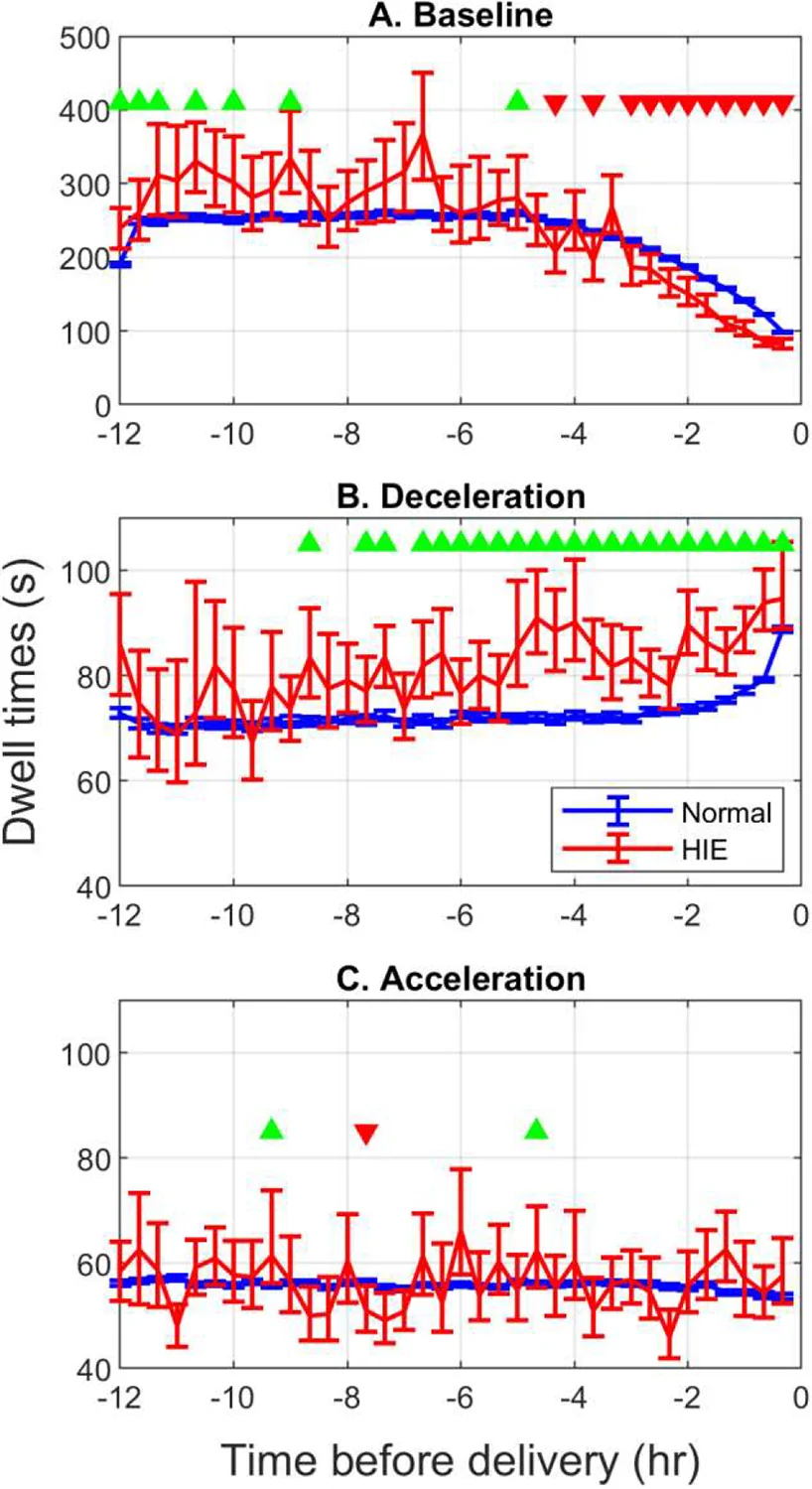
The 50^th^ percentile of the expected value of the (A) baseline, (B) deceleration, and (C) acceleration dwell time for the normal (blue) and the HIE (red) groups as functions of time before birth. The error bars show the 2.5^th^ – 97.5^th^ percentile range. Triangles indicate significant differences according to the Kolmogorov-Smirnov test and whether the estimates were larger (green upwards) or smaller (red downwards) for HIE than for controls.

**Figure 6.**
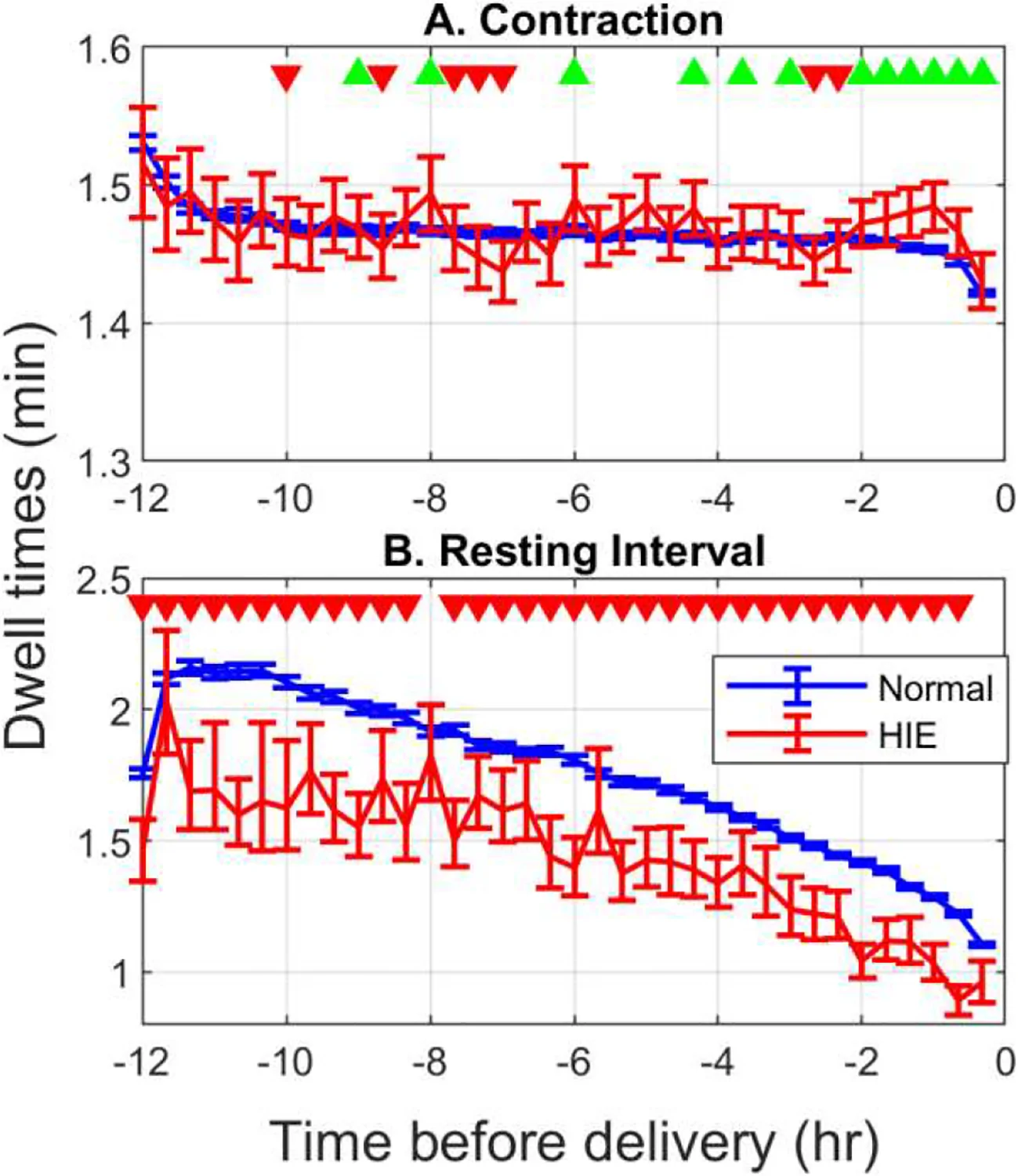
The 50^th^ percentile of the expected value of the (A) contraction and (B) resting interval dwell time for the normal (blue) and the HIE (red) groups as functions of time before birth. The error bars show the 2.5^th^ – 97.5^th^ percentile range. Triangles indicate significant differences according to the Kolmogorov-Smirnov test and whether the estimates were larger (green upwards) or smaller (red downwards) for HIE than for controls.

## Clinical Relevance—

Our results revealed statistically significant differences in FHR/UP patterns between the normal and HIE groups in the hours before birth. This indicates that features derived using MCSMMs may be useful in a machine learning framework to detect infants at increased risk of developing HIE, allowing preventive interventions.
